# Effect of Inoculant Alloy Selection and Particle Size on Efficiency of Isomorphic Inoculation of Ti-Al

**DOI:** 10.3390/ma11050666

**Published:** 2018-04-25

**Authors:** Jacob R. Kennedy, Bernard Rouat, Dominique Daloz, Emmanuel Bouzy, Julien Zollinger

**Affiliations:** 1 Department of Metallurgy & Materials Science and Engineering, Institut Jean Lamour, Université de Lorraine, Campus ARTEM, Allée André Guinier, F-54011 Nancy, France; jacob-roman.kennedy@univ-lorraine.fr (J.K.); bernard.rouat@univ-lorraine.fr (B.R.); dominique.daloz@univ-lorraine.fr (D.D.); 2 Laboratory of Excellence on Design of Alloy Metals for low-mAss Structures (DAMAS), Université de Lorraine, 57073 Metz, France; emmanuel.bouzy@univ-lorraine.fr; 3 Université de Lorraine, CNRS, Arts et Métiers ParisTech, LEM3, F-57000 Metz, France

**Keywords:** titanium aluminides, grain refinement, solidification, inoculation

## Abstract

The process of isomorphic inoculation relies on precise selection of inoculant alloys for a given system. Three alloys, Ti-10Al-25Nb, Ti-25Al-10Ta, and Ti-47Ta (at %) were selected as potential isomorphic inoculants for a Ti-46Al alloy. The binary Ti-Ta alloy selected was found to be ineffective as an inoculant due to its large density difference with the melt, causing the particles to settle. Both ternary alloys were successfully implemented as isomorphic inoculants that decreased the equiaxed grain size and increased the equiaxed fraction in their ingots. The degree of grain refinement obtained was found to be dependent on the number of particles introduced to the melt. Also, more new grains were formed than particles added to the melt. The grains/particle efficiency varied from greater than one to nearly twenty as the size of the particle increased. This is attributed to the breaking up of particles into smaller particles by dissolution in the melt. For a given particle size, Ti-Al-Ta and Ti-Al-Nb particles were found to have a roughly similar grain/particle efficiency.

## 1. Introduction

Titanium aluminum alloys are an interesting material for aerospace applications. Their high temperature oxidation resistance, and high specific strength make them particularly promising [1]. Recently, titanium aluminides have replaced nickel based superalloys in the last low pressure stage of the GEnx turbine engine. In order to increase their usefulness in turbines, their properties must be improved for high temperature (>800 °C) applications. Grain refinement is one method to improve the properties of a material, however, the current methods of grain refinement of Ti-Al alloys by inoculation can negatively affect ductility [2]. The Ti-Al alloys of interest for aerospace applications normally fall on the peritectic plateau of the phase diagram at 44–48% Al. The solidification path for alloys in this composition range is complex, and can include multiple phase changes from liquid to room temperature [3]. After solidification the solid state transformations have known orientation relationships, such as: β to α (orientation relationship (110)_β_ // (0001)_α_ [4]) and α and γ (orientation relationship (0001)_α_ // (111)_γ_ [5]). Grain refinement can be attempted during any of these phase transformations, however, attempts to grain refine by using the γ massive transformation during heat treatment has been shown to require fast cooling rates, which result in deformations, making the application of the method difficult [6,7]. The location of grain refinement during processing that is most interesting is then the initial solidification of β grains from the liquid.

Inoculation is a method of grain refinement by increasing the number of grains that were formed during solidification [8]. This is achieved by increasing the number of nucleation sites present in the melt for the solid to form from [9]. Powders can be added, which are effective nuclei or alloying additions can be made, which will form precipitates in the melt that act as nuclei [10]. The most common method of inoculating Ti-Al alloys is via boron additions [11]. Relatively small amounts of boron are added to the melt and titanium boride (TiB) or diboride (TiB_2_) precipitate and act as nucleants. These precipitates can nucleate α or β phases, depending on alloy composition, and have been found to have orientation relationships with both phases, indicating a good lattice mismatch between the inoculant and nucleated phases [12]. These borides have also been found to be thermodynamically stable in the Ti-Al melt [13]. Boron additions are especially effective at low levels, where they can cause interdendritic nucleation of α phase grains [14]. However, the borides that form may have flake [15] or needle like [11] morphologies in the as-cast state. These morphologies are detrimental to the final mechanical properties of the alloy, and can result in decreased creep resistance and tensile strength, leading to brittle fracture [2]. This illustrates the need for an inoculant in the TiAl system that is more ductile to prevent this embrittlement of the as-cast material.

Nucleation via inoculation is commonly represented by the spherical cap heterogeneous nucleation model [8]. This model is based on a reduction of activation energy when compared to homogenous nucleation that is caused by the presence of solid surfaces that act as nucleation sites. This reduction of activation energy depends on many factors, such as the interfacial energies between the liquid, solid, and nucleus [10], as well as the shape of the solid (i.e., flat, convex, concave) [16]. This model accurately describes less efficient nucleants, however, in the case of inoculants with higher efficiencies, nucleation can be described as atom by atom adsorption onto the solid surface [17] or by particle wetting [18]. Even in these more efficient cases, there is still an energy barrier for nucleation to overcome in order for solidification to progress. A novel solution to this problem was proposed by the authors, called isomorphic self inoculation, where rather than having the inoculants act as centers for nucleation particles are used, which can act instead as centres of growth, bypassing the nucleation step and requisite energy barrier [19]. In this case, as there is no energy barrier to surpass for solidification to progress, each particle that is added can be active in solidification and be the center of a new grain. A similar method of grain refinement was previously investigated by Bermingham et al., for a Ti alloy by pouring the melt over pure Ti powders and into a mold [20]. 

In order to develop inoculants that function in this manner, four design criteria were proposed: (I) Phase and lattice matching, (II) Thermal and diffusive stability in the melt, (III) Good usability factors, and (IV) No negative effects on final casting. The first two criteria are the phenomenological factors determining if the inoculant can successfully act as an isomorphic inoculant. The final two criteria are related to processing and implementation; if the inoculant is a viable alternative to other inoculants. The critical phenomenological basis for isomorphic self inoculation is phase and lattice matching. By introducing solid particles to the melt which are the same phase as the solidifying alloy with similar lattice parameters the nucleation stage of solidification, and its requisite energy barrier can be circumvented by the direct growth of the particles [19]. Rather than having new solid matrices form on the particles the matrix of the particle itself grow as the melt solidifies epitaxially upon it. The second phenomenological criteria for the design of an isomorphic inoculant is to ensure stability in the melt. Particles that match the phase and lattice of the bulk must survive long enough in the melt to affect solidification in order to be viable isomorphic inoculants. The stability of the particles in the melt does not have to be absolute. Some dissolution or melting is acceptable if the particles can survive past thermal equilibrium with the melt and participate in solidification. The first non-phenomenological design criteria for an isomorphic inoculant is its good usability. This refers to the ease of which the inoculant can be produced and implemented, as well as its behaviour in the melt. These factors may or may not fundamentally affect whether the inoculant can function as designed, but rather affect if it can realistically grain refine a casting. This includes such factors as density of the particles causing them to float or to settle in the melt, the ability of the particles to be easily manufactured, whether the procedure of adding the particles to the melt is difficult and any other factors that affect the actual usage of the particles. The final criteria is that the inoculants do not negatively affect the final properties of the material. It is important to look at the elaboration process as a whole and to ensure that the isomorphic inoculants do not negatively affect either the casting or the final part. This is important for Ti-Al alloys that are normally used in high temperature applications where even if the as cast part may have ideal grain size with no precipitates solid state transformations may occur causing phases, such as σ in the Ti-Al-Ta system [21] or ω in the Ti-Al-Nb system [22], to form which are detrimental to mechanical properties. If any particles do remain heterogeneous in the matrix they must not be too brittle to negatively affect the ductility. 

## 2. Inoculant Selection

In order to determine if the system is suitable and if a suitable composition exists within it for an isomorphic inoculant, each of the four factors above were evaluated. In order to do so, available information on the alloys was required on the thermodynamic description of the system, diffusivity of the alloying elements in β-Ti, the lattice parameter of the alloy, as well as their densities. The thermodynamic description is necessary to ensure that the particles are thermally stable in the melt, as well as exist in the matched phase field with the solidifying bulk phase at the melting temperature of the bulk alloy. The diffusivity of the alloying elements in the inoculant are important for their diffusive stability in the melt. The lattice parameter of the alloy is necessary to check the lattice mismatch with the bulk alloy. The alloy density at high temperature is important for usability issues, as well as at room temperature for calculating the number of inoculants that are introduced during casting trials. In these trials, a base alloy of Ti-46Al (at %) was used. From the phase diagram, the solidification phase for this alloy can be seen to be β-Ti with a melting temperature of ~1540 °C [3]. This means that the inoculant alloys must have melting temperatures that are greater than 1540 °C and be in the β-Ti phase field of their systems at high temperature. In addition to the minimum melting point, the base alloy selection also effects how quickly the inoculants will dissolve in the melt. In order to evaluate the survivability of the inoculants, the diffusion species in the particles at high temperature were considered. Assuming that the particles and the particle/melt interface reach thermal equilibrium with the melt their dissolution should be controlled by the slowest diffusing species out of the particles and into the melt, in order to maintain the conservation of mass across the interface. Slower diffusion rates should correspond with slower diffusion of the inoculant particles and better survivability in the melt.

An equation has been fit to alloys in the Ti-Al-Nb-Ta system to find their lattice parameter at high temperature [23]. The composition of the alloy in atomic percent (X_i_) and the temperature in degrees Celsius (T) are taken as inputs and the lattice parameter is the output (α_β_) in nm, as can be seen below:

(1) $$\alpha_{\beta }=\alpha_{\beta }^{\mathrm{Ti}\left(\mathrm{Pure}\right)}+\sum_{j=1}^{k}e_{i}X_{i}+b$$

where α_β_^Ti(Pure)^ is the beta Ti lattice parameter at 0 °C in nm. Using reported lattice parameters for pure Ti at different temperatures, first, the equation could be solved for b, and then each coefficient, respectively, using reported lattice parameters at different compositions. To find the lattice parameter in nm with component compositions in atomic fractions (X_i_) at a given temperature in °C (T), the equation was determined to be [23]:

(2) $$\alpha_{\beta }=0.328-7.4\times 10^{-5}\left(X_{\mathrm{Al}}\right)+1.23\times 10^{-4}\left(X_{\mathrm{Nb}}\right)+4.6\times 10^{-4}\left(X_{\mathrm{Ta}}\right)+7.4\times 10^{-5}\left(T\right)$$

The density of the alloys at high temperature is important as the inoculants should have densities as close as possible to the bulk alloy. If the density of the inoculant is significantly far from that of the bulk alloy problems may arise with segregation of the particles in the melt. A perfect match in densities is not critical in these casting trials as an induction furnace was used, which stirs the melt, helping to avoid segregation. The density of each alloy at high temperature was calculated using the molar mass of the constituent elements and the calculated lattice parameter. Since β-Ti is a Body Centered Cubic (BCC) structure a unit cell contains two atoms. The mass of the unit cell of the alloy (m^a^) can then be calculated using the molar mass of each element (M_i_) and its amount in the alloy by atomic fraction (X_i_) and Avogadro’s number (N). Since a BCC structure is cubic the volume of a unit cell is simply the cube of the lattice parameter at any temperature (a_β_), and the density of an alloy at a given temperature (ρ_β_) can be calculated with:

(3) $$\rho_{\beta }=\frac{m}{V}=\frac{2\sum^{\text{​}}M_{i}X_{i}}{N{\left(a_{\beta }\right)}^{3}}$$

**Figure 1:**
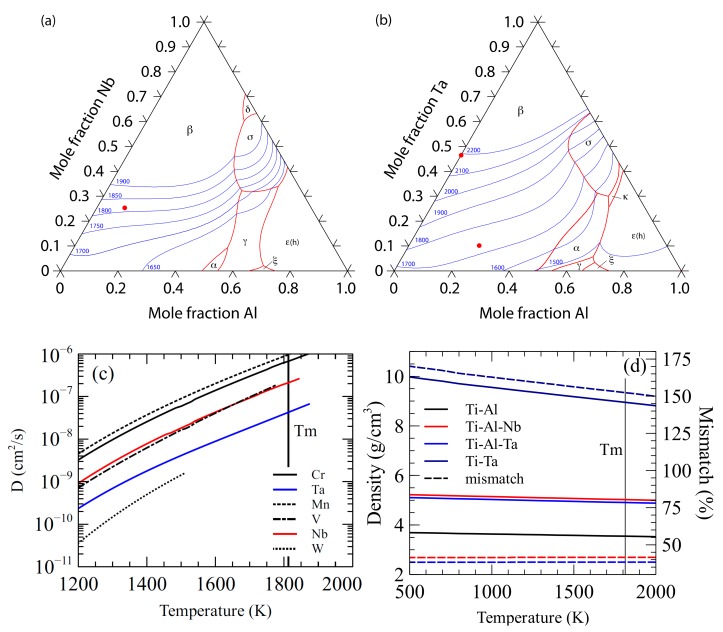
(**a**) Liquidus surface map for the Ti-Al-Nb System (data from ref. [22]), (**b**) Liquidus surface map for the Ti-Al-Ta system (data from ref. [21]) (**c**) Calculated diffusion rates of some refractory metals in high temperature β-Ti (data from ref. [24]), and (**d**) calculated densities (solid lines) and difference from bulk (dashed lines) with temperature.

Two refractory metal additions were proposed to form inoculant alloys that would function as isomorphic inoculants, Nb and Ta, their liquidus surface diagrams are shown in Figure 1a,b, respectively. Both of the ternary systems have large β solidifying fields with melting points above the bulk alloy. A plot of the diffusivity of common refractory metals in β-Ti is shown in Figure 1c, where Ta and Nb are shown to diffuse slowly at high temperature when compared to other refractory metals [24].

The first system that was investigated for potential isomorphic self-inoculants was the ternary Ti-Al-Nb. Nb is a beta stabilizing element in the Ti system [25] with a relatively high melting point of 2415 °C [26]. A balance between lattice mismatch, stability, and density was sought to find an ideal inoculant. An inoculant alloy of Ti-10Al-25Nb was selected. It exists as the β-phase at high temperature, has a high melting point (1800 °C [27]), has a small lattice mismatch with the base alloy (<2%), and the Nb content should provide some diffusive stability. The density of the alloy is greater than that of the bulk by roughly 40%, however, the thermal and diffusive stability, along with the low lattice mismatch, make it a good candidate for an isomorphic inoculant.

The second system that was proposed for an isomorphic self-inoculant was Ti-Al-Ta. Ta has a higher melting point (3000 °C [26]), but it is denser than Nb. Again, a compromise was sought between stability, density, and lattice mismatch. The proposed alloy of Ti-25Al-10Ta was selected as a suitable inoculant for Ti-46Al. It has a small lattice mismatch (<2%) with the base alloy, a higher melting point (1725 °C), and it exists as β-Ti at high temperature. The relatively slow diffusion of Ta in β-Ti should provide the inoculant with good diffusive stability.

A third inoculant system was proposed to maximize both the thermal and diffusive stability at the expense of lattice and density mismatch. The alloy that is needed to maintain a relatively small lattice mismatch, but the maximization of stability was given greater priority than minimization of mismatch. To this end, the binary Ti-Ta system was investigated and Ti-47Ta selected, as this was the maximum Ta content possible without risking formation of the σ phase after interaction with the Ti-46Al bulk alloy. Ti-47Ta has a higher lattice mismatch (<5%) that the other inoculant alloys, but it remains low enough to be a suitable inoculant. Its high melting point (2200 °C) and the relatively slow diffusion of Ta should make it the most stable of the inoculants that was proposed for the base metal. The density of the alloy is much greater than the base alloy so the usability of the inoculant may be decreased and its behavior in the melt may be unideal, a comparison of the alloy densities is shown in Figure 1d, and a summary of the inoculant alloy properties is given in Table 1.

## 3. Materials and Methods 

Bulk inoculant alloys were fabricated from commercially pure elements in an induction heated cold crucible apparatus. Before melting, the chamber was pumped down to a vacuum of 10^−3^ mbar, and then an Ar flux was applied, allowing for processing to occur at atmospheric pressure without the risk of oxidation. The alloying element with the highest melting point was used as the base for the alloying with other elements added consecutively in order of their melting point from highest to lowest. Once bulk alloys had been fabricated, it was necessary to turn them into powders that could be used for inoculation. This was achieved by drilling into the ingots to produce chips, the majority of which were in the order of mm, too large to be implemented in our laboratory apparatus. In order to reduce their size the powders were cryomilled in a Retsch CryoMill (Retsch–Verder group, Haan, Germany). Cryomilling was chosen as the low temperature would embrittle the particles, as well as prevent them from agglomerating or sticking. Drillings were added to the milling container along with steel balls with a ratio of one steel ball for every gram of powder. The apparatus ran liquid nitrogen around the milling container to maintain a temperature that is near −196 °C. To obtain particles of different sizes drillings of each alloy were milled for different lengths of time, ranging from 1.5 to 11 h. Initially, powders were milled in air, however to minimize the potential effects of oxidation the Ti-Ta powders were milled under Ar, and a further sample of Ti-Al-Nb was milled in Ar as well to compare with those milled in air. Once the powders had been prepared, it was necessary to characterize them. The size of the powders was important to know since it is of critical importance for diffusive stability. Additionally, in order to calculate the number of particles that were added in an inoculation trial, the size distribution must be known. The size distributions were determined by image analysis of the particles taken by Scanning Electronic Microscopy (SEM) (FEI–Thermo Fisher Scientific, Hillsboro, OR, USA). The particle diameters that are reported are from equivalent circular areas from the projected two-dimensional (2D) areas of the particles. The resultant D50 and spans ([D90-D10]/D50) of the distributions are shown in Table 2.

In order to test the ability of the alloys as inoculants, ingots (~40 g) of TiAl were produced both with and without inoculation, and their grain sizes were compared. To inoculate the samples the previously produced powders were formed into pellets by cold pressing the inoculant particles and with an equal amount of aluminum powder (1 g each). These pellets were held by vacuum on a small quartz tube above the Ti-Al alloy. Once the Ti-Al was fully melted the vacuum was released and the pellet fell into the molten alloy. The inoculants remained in the melt for 20 s before the furnace was turned off and the alloy was allowed to solidify. This time period corresponds to the time for the pellet to disperse into the melt and the molten droplet to return to a regular shape within the induction field. The ingots were then bisected, polished, and etched with Krolls reagent. In addition to the inoculation trials, an ingot was produced in the same manner without any inoculants to determine the reference conditions. A further ingot was also produced by increasing the interaction time with a Ti-Al-Nb distribution and remelting after the inoculation three times in order to dissolve all of the inoculants to determine the effect of the Nb alloy addition on the conditions, independently of the inoculation. 

## 4. Results

**Figure 2:**
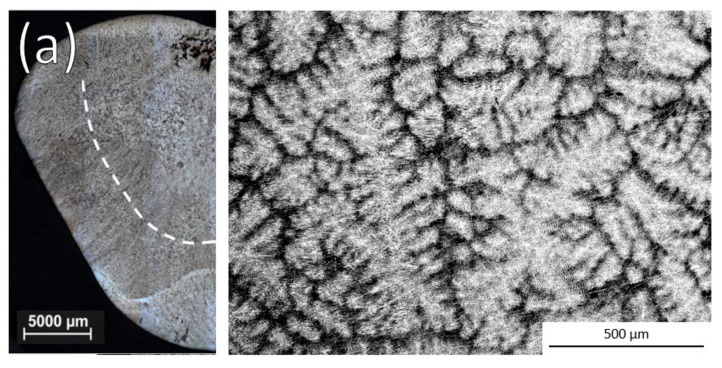

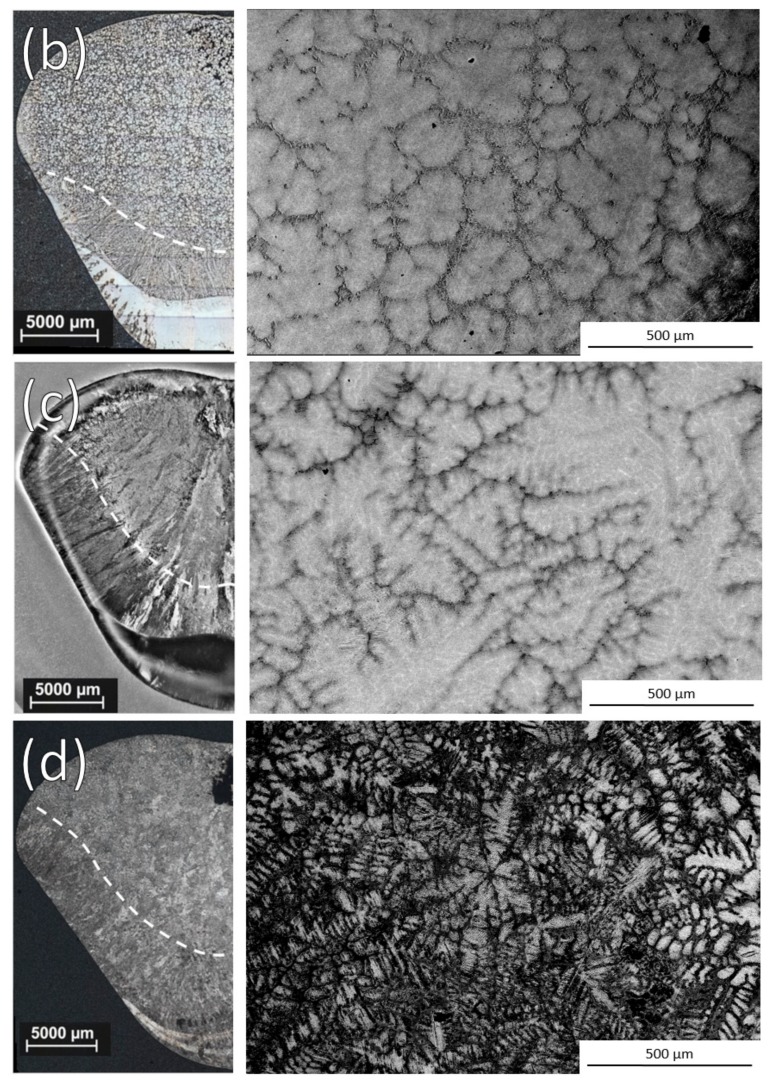
Optical ingot cross sections and SEM BSE images of equiaxed zone after (**a**) No inoculation and inoculation with 3 h cryomilled particles of (**b**) Ti-Al-Nb, (**c**) Ti-Al-Ta, and (**d**) Ti-Ta.

Example optical images of the ingot cross sections and SEM Back-Scattered Electrons (BSE) images of the equiaxed microstructure for a non-inoculated ingot and ingots that were inoculated with each alloy are shown in Figure 2. Whether or not grain refinement was observed with each size distribution of the alloys is shown in Table 2.

Measuring the size of dendritic grains consistently and accurately is non-trivial. Depending on the plane of the ingot being viewed, dendritic grains may appear very differently, conventional grain size measurement using the line-intercept or a similar method is not possible. In order to ensure that the grain size reported was reflected to be as close to the real grain size as possible, only grains that appeared to be cut in an exact cross section perpendicular to four primary arms were measured. The linear lengths from opposing tips were measured for two pairs of tips as close to perpendicular to one another as possible were used to characterize the size of such grains. These measurements were taken across as many grains as possible in order to obtain an average grain size. Grains were identified and measured manually. This method minimizes the total number of grains measured, since only grains with a near complete cross section are measured, but ensures that each measurement relates precisely to a real dimension of a grain. In order to have comparable results between samples the same method was used in all cases, even if there were globular shaped grains which could have their size evaluated effectively with other methods. The equiaxed fraction of the ingots was also measured. These measured values for the successful inoculation trials along with the final Al contents of the ingots are shown in Table 3.

**Figure 3:**
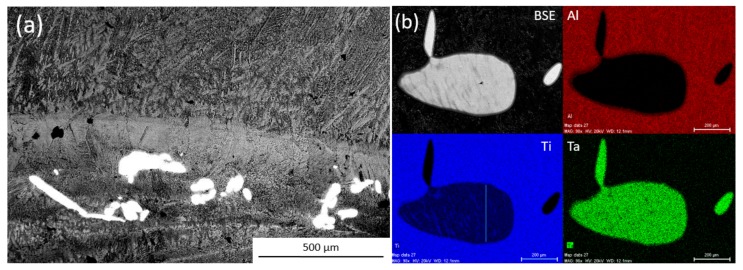
(**a**) BSE images of bottom region of ingots inoculated with Ti-Ta and (**b**) Energy Dispersive Spectrometer X-ray diffraction (EDX) maps of particles found.

While the Ti-Ta particles were designed to have the greatest stability in the melt none of the inoculation trials that used them were successful at reducing the as-cast equiaxed grain size. While no particles were found in the equiaxed region of any of the inoculated ingots, some Ti-Ta particles were found to have survived processing. These particles were found in the bottom of the ingot, either in the columnar zone or in the semi-solid cap at the bottom of the ingot. Figure 3 shows an SEM BSE image from this semi-solid bottom cap/columnar zone of an ingot inoculated by Ti-Ta. Energy Dispersive Spectrometer X-ray diffraction (EDX) analysis was performed on the particles that were found in the ingots inoculated with Ti-Ta, an EDX map of a representative particle from the 1.5 h inoculation trial is also shown. The particles were confirmed to contain both Ti and Ta with compositions near expected for undissolved inoculant particles (Ti-47Ta). 

It can be noted that the mapped particle has a length over 600 μm and a width of 300 μm, this is significantly larger than the both the D50 (70 μm) or D99 (334 μm) for the distribution. The BSE image of the particle shows a somewhat banded structure that can also be seen particularly well in the Ti composition map. This indicates that it exists as a single particle, rather than an agglomeration of smaller particles. This is interesting as such large particles were not detected by SEM image analysis. A single particle of such size has a mass equal to 0.06 wt % of the distribution, when compared to 0.02% for a particle identical to the D99 or 0.0002% for the D50. It then does not take very many large particles existing in the distribution to severely decrease the number of particles that are available for inoculation This could help to explain why the Ti-Al trials did not show any grain refinement, as super large particles unaccounted for in the distributions reduced the number of active particles that are added to the melt. The small number of these particles do not lend well to their detection by imaging methods.

In addition to the super large particles shown some smaller particles of Ti-Ta were also found in the solidified ingots that were within the size range of the distributions measured. However, all of these smaller particles were found in the columnar zone of the ingots, or along with the super large particles in the bottom cap. Particles in these regions did not help to form equiaxed grains, as the region that they are in has no equiaxed grains. The question is then how the Ti-Ta particles were deposited in this region when there are no Ti-Al-Ta or Ti-Al-Nb particles that were detected in the same regions of their respective ingots. The fluid flow of the molten Ti-Al alloy should be the same in each inoculation trial, as the processing conditions remain constant, however, the particles may behave differently from one another in the melt as they have different sizes and densities. It is difficult to calculate how the particles move in the melt as the induction field induces complex three-dimensional fluid flows into the molten drop which cannot be easily approximated. The solid bottom cap is humped in the center of the ingot and has a shape matching that of the fluid rolls. Many of the TiTa particles are found in this central humped region, indicating that, rather than being carried by the fluid flow and dispersed throughout the ingot, they have been ejected from the flow and settled in this region where they are not useful for grain refinement of the equiaxed zone. 

The ingots inoculated with TiTa also showed dendrite morphologies that matched the α phase (hexagonal) rather than β (cubic). This could be the result of oxygen pickup during the high temperature processing that is required to produce the inoculant alloy. β-TiTa has a large oxygen solubility so that this transition to α was not seen in the bulk inoculant alloy, however, the oxygen solubility is much less in β-TiAl [30]. An increase in oxygen content of the alloy may then also be poisoning its effectiveness at grain refinement as if the solidification phase has changed from β to α the lattice and phase matching for which the inoculant was designed no longer exist.

It is then likely that a combination of these factors lead to the TiTa particles being ineffective. The high density of the particles results in the particles settling to the bottom of the ingot removing the particles from the equiaxed zone where they are useful for decreasing the equiaxed grain size, ensuring that the inoculants are ineffective. The presence of superlarge particles that account for a large portion of the inoculant mass added to the melt reduces the number of inoculants added to the melt. The inoculants that were added to the melt may be poisoning it against them by adding oxygen and inducing a change of the solidification phase from β to α. 

All of the inoculation trials with Ti-Al-Nb and Ti-Al-Ta particles were successful at both decreasing the equiaxed grain size and also increasing the fraction of the equiaxed zone in the ingots. The magnitude of the grain refinement and the enlargement of the equiaxed zone depended on the alloy and particle size distribution that was used. It can be seen that the equiaxed grain size tended to decrease as the milling time (Table 3) increased, except in the case of the 6 h milled sample, which has a larger grain size than expected, however the grain size is still reduced when compared to the reference or solutal samples. Milling in Ar resulted in larger equiaxed grains than particles that were equivalently milled in air. The same was true for the resultant equiaxed fractions; milling in Ar was less effective at increasing the equiaxed zone when compared to air. The equiaxed fraction stayed relatively constant between the air milled particles with the exception of the 11 h milled sample that had a large unmelted Ti particle that was inherited from primary ingot elaboration, and larger bottom cap that was present in the cross section. The Ti-Al-Ta particles reduced the grain size roughly the same amount as equivalently milled Ti-Al-Nb particles, however, the three hour Ta containing particles did not increase the equiaxed fraction as much as the others.

It was also important to know the number of inoculant particles added in each inoculation trial. Since the mass of the inoculant particles that were added was measured, it could be used along with the size distributions that were obtained and the calculated densities to determine the number of particles added. This was done assuming the particles were spherical and that all the particles in each size range had a diameter exactly the average of the maximum and minimum values of the range. Using the measured mass of inoculant (≈1 g) and the calculated density of the inoculant alloy, a total volume of inoculant introduced could be found. The particles were also assumed to be spherical when calculating their sized from projected 2D areas, however, if an ellipsoid diverges from a spherical shape (with a constant average radius), its volume will decrease. The calculated number of particles then may be underrepresented if the particles vary significantly from spheres.

The size range of the two Ti-Al-Ta distributions that were tested are between the 9 and 11 h milled Ti-Al-Nb particles. The two particle sizes both reduce the grain size somewhat less than that of the Ti-Al-Nb 11 h milled distribution, which has the closest D50 to the Ti-Al-Ta distributions used. However, when the number of particles introduced is considered the grain sizes are much closer to the trend of the Ti-Al-Nb particles. The Ti-Al-Ta trials introduced more particles than when the 9 h milled Ti-Al-Nb particles were used, but less than with 11 h Ti-Al-Nb particles. As the same mass of inoculant is introduced in each trial, particle size and the number of particles that were introduced are directly correlated. If we compare Ti-Al-Nb (11h) and Ti-Al-Ta (9 h) with roughly the same median particle size, we can see that the refining power of the Ti-Al-Nb is greater because the number of particles is higher, indicating that the critical parameter is not the size of the particles, but rather the number of particles that are introduced. This is in direct contrast to traditional inoculation, where the size of the particles that are introduced is the most critical as larger particles can nucleate new grains at smaller undercoolings [31].

## 5. Discussion

**Figure 4:**
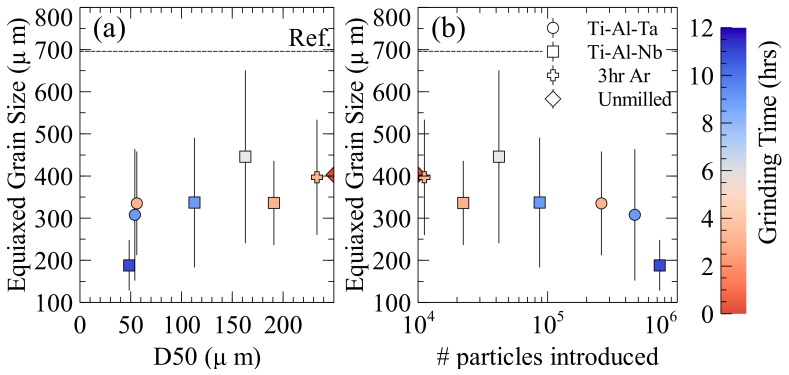
Influence of (**a**) particle size and (**b**) number of particles introduced on equiaxed grain size. The error bars correspond to the standard deviation of grain size measurements.

The resultant grain size as powder size and number of particles introduced to the melt increase can be seen in Figure 4a,b, respectively, for the Ti-Al-Nb and Ti-Al-Ta inoculation trials. The reported error is the standard deviation of the grain size measurements. Since only equiaxed dendrites with nearly complete cross sections are measured, the number of grains that are measured is limited. Ingots with larger grain sizes will have fewer grains cut in cross section that may be measured, and thus the standard deviation of their measurements will be larger, this is also true of structures that are more dendritic compared to globular. This may obscure some of the trends observed, but it ensures that the reported grain sizes are representative of real structures within the ingots. When evaluated based on the size of the particles introduced the Ar milled sample does not appear to behave significantly differently from those that are milled under air. While milling under Ar for 3 h resulted in larger particles than the same time under air, the resultant grain size and equiaxed fraction are roughly what would be expected for particles that were milled under air with the same size. The Ar milled powders also behaved as the particles milled in air with respect to the resultant equiaxed fraction. The six hour milled sample (D50 = 160 μm) resulted in a larger grain size than expected. Since each bulk alloy was produced individually some variation in the composition is to be expected. This variation may account for the six hour inoculations apparent deviation, as it has the leanest Al composition of all the inoculated ingots. This is important since the grain size has been shown to increase in ingots produced with similar processing as Al content decreases due to the changes in the dendrite fragmentation mechanism [32]. This means that, while the grain size may be larger than expected in the ingot that is inoculated with the six hour milled particles, the reduction in grain size due to the inoculation may be greater than shown, since the bulk alloy would have had a larger grain size than the measured reference sample due to its decreased Al content.

**Figure 5:**
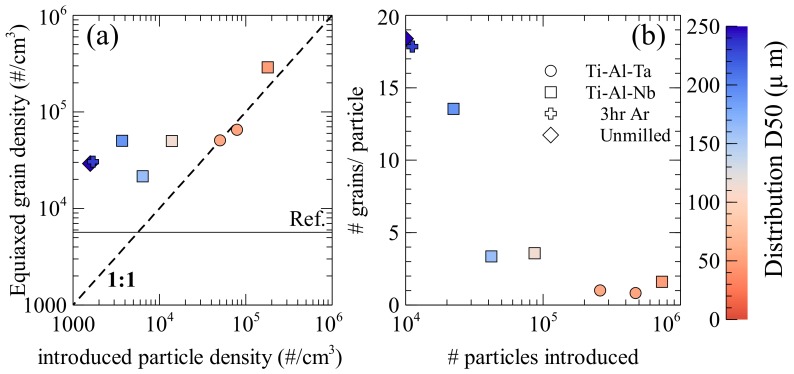
(**a**) Influence inoculant particle density assuming particles only present in the equiaxed zone and (**b**) Relationship between # of grains formed by each inoculant and number of particles introduced if they are only in the equiaxed zone.

Using the average equiaxed grain size, the grain density in the equiaxed zone (#/cm^3^) can be compared to the density of particles that are added to the melt (#/cm^3^), as shown in Figure 5a. This assumes that the all of the particles that were added were evenly distributed in the equiaxed zone. This gives the most pessimistic results of inoculant efficiency, as this is the maximum density the particles could have during solidification, if they were evenly dispersed throughout the ingot their density in the equiaxed zone would be reduced. It is also assumed that no particles completely dissolve, meaning that the same number of particles is present during solidification as was added to the melt. In Figure 5a the dashed line indicated a 1:1 ratio, where each particle would add a single grain to the solidified ingot. In every case, except the 9 h Ti-Al-Ta (which still close to this 1:1 ratio), the grain density is larger than the introduced particle density, which means that more grains were formed than particles that were added to the melt. Moreover, it can be seen that when the number of particles introduced exceeds 5 × 10^4^ particles/cm^3^ the efficiency tends towards 1. When fewer particles are added the grain size does not change drastically, rather the efficiency of the particles for isomorphic inoculation increases. This is in strong contradiction to traditional inoculation where the number of grains formed is significantly less than the number of particles that were added to the melt [33,34]. The authors previously proposed that this drastic increase in efficiency was due to the suppression of the nucleation step, allowing for direct particle growth [19] and the increase above a 1:1 ratio of particles to grains due to a process of particle breakup. The effect of particle size on particle break-up is estimated in Figure 5b, where the evolution of the number of equiaxed grains that were formed by single particle is compared with the number of introduced particles, the distribution D50s are indicated. There is a sharp increase in number of grains that were formed per particle with large particles when compared to small particles. The smaller particles (~50 µm) result in one or two grains being formed per particle, with both Ti-Al-Nb and Ti-Al-Ta, while the Ti-Al-Nb particles of intermediate size (100–170 µm) have 2–5 grains formed per particle and the Ti-Al-Nb particles larger than 170 μm result in more than 10 grains formed per particle. It is then possible that the close diffusivity and processing method results in particles that break up in roughly equivalent manners between the two alloys. The difference in effectiveness may then be attributed to the mechanical properties of the particles that behave differently under milling resulting in different distributions, and a greater difference in particle number for roughly equivalent particle D50s. The general efficiency of the inoculation then depends on the number of particles that were introduced, however, the particle size distribution must also be taken into account, since if the particle size is large, significant particle breakup may occur, while when the particle size is low, the breakup factor tends towards one. This means when the introduced particle size is low the correlation between number of particles introduced and the number of grains formed is stronger than with large particles where the distribution may be more affected by interaction with the melt. 

**Figure 6:**
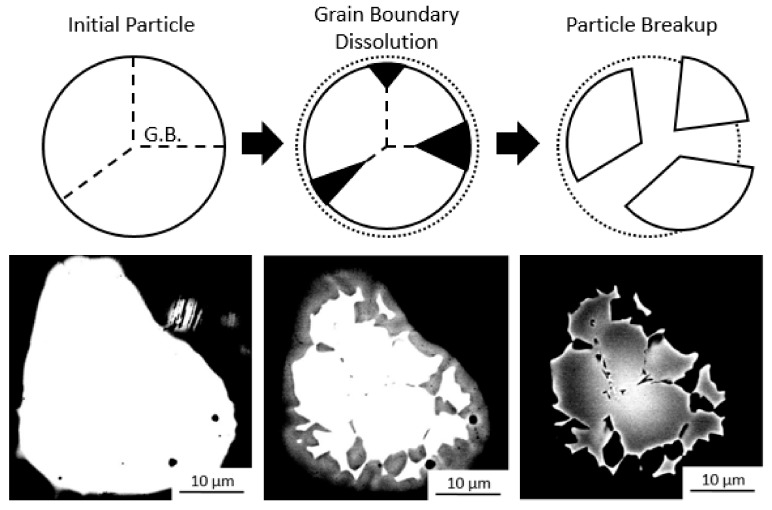
Schematic of particle breakup process: whole polycrystalline particle, preferential grain boundary dissolution, particle breakup, and BSE micrographs of Ti-Ta particle in solidified ingot demonstrating particle breakup by adjusting imaging contrast.

The particle break-up mechanism was observed in a Ti-Ta inoculated ingot, and is shown in Figure 6, together with a schematic representation. In Figure 6, the bright Ta rich regions are portions of a particle which are in the process of breaking up while the dark regions are the bulk alloy. By adjusting the brightness and contrast when obtaining the images, the particle shape can be seen to vary from a monolithic single particle to a collection of smaller particles that are separated by regions of lower Ta concentration. This may occur by preferential dissolution [35] and/or impingement of the liquid along the grain boundaries of the particle [36]. Such impingement or wetting of a liquid like phase along grain boundaries has been observed in-situ along Al grain boundaries by liquid Ga [37]. Such wetting may occur if the grain boundary energy is higher than that of two solid liquid interface [38] i.e., $\sigma_{\beta /\beta }>2\left(\sigma_{\beta /\ell }\right)$, where σ is the surface energy. In this case the liquid will be fully wet along the grain boundary, which would permit the separation of the particles in the melt along the grain boundaries. The dark liquid can be seen to ingress along preferential paths, resulting in the breakup of the particle. As the size of the particles increases, so does the number of grains that are formed by each particle. This could be due to an increased number of cracks or grains present in each large particle when compared to the smaller ones, as they have been milled for less time. These cracks which on further milling result in particle breakup during milling may act as preferential dissolution paths in the melt resulting in a larger breakup effect during inoculation. This would also explain the decrease in the number of grains that are formed by each particle as the number of particles introduced increases. The smaller particles, which were introduced in more plentiful numbers, were also more likely to dissolve completely and less likely to break up into more particles. Again, the breakup and dissolution of particles likely reduces the global maximum and increases the global minimum number of particles that are present on solidification, the number and the size of particles introduced to the melt are indicative of, but not entirely representative of, the distribution present on solidification.

## 6. Conclusions

In summary, the efficiency of isomorphic inoculation was evaluated based on inoculant alloys selection, accounting for thermal and diffusive stability, particle size distribution, and particle density. The results presented here show that, within this experimental set-up, the most critical parameter is the density difference between the liquid alloy and the inoculant powder. High density differences lead to settling of inoculant particles and prevents grain refinement. In contrast, the diffusive stability of the particles was found to be less critical and only affect the observed as-cast grain size slightly when the other parameters were similar.

Isomorphic inoculation relies on epitaxial growth of the solidifying alloy on inoculant particles. The critical parameter for isomorphic inoculation was proposed to be the number of inoculant particles in [19], as opposed to the particle size distribution as in classical inoculation processing. In this work, it has been shown that particle size distribution also contributes to the efficiency of the isomorphic inoculation process. It was found that larger particle size distributions were more efficient for grain refinement of Ti-Al alloys, this increase is attributed to the trend of the particles to break up into multiple smaller particles by dissolution in the melt. This phenomenon is more apparent if the initial particles are large, showing that there is a critical particle size below which dissolution occurs faster, leading to a decrease in surviving particles, and thus a decrease in inoculation efficiency. 

## Figures and Tables

**Table 1:** Summary of inoculant alloy properties.

Alloy	α (nm) (PW)	T_m_ (°C) [27]	ρ (g/cm^3^) (PW)		Slowest Diffusing Species	D_β_-Tracer (cm^2^/s) [24]	D_β_ Interdiffusion (cm^2^/s)
			25 °C	1540 °C			
Ti-46Al	0.330	1540	3.71	3.55			
Ti-10Al-25Nb	0.335	1800	5.25	5.02	Nb	1.40 × 10^−8^	1.02 × 10^−8^ [28]
Ti-25Al-10Ta	0.336	1725	5.13	4.91	Ta	5.63 × 10^−9^	1.22 × 10^−8^ [29]
Ti-47Ta	0.345	2200	10.14	8.95	Ta	5.63 × 10^−9^	2.34 × 10^−10^ [29]

**Table 2:** Inoculation Trial Conditions.

	Cryo-Milling		Particle Distribution Parameters		
Alloy	Time (h)	Atmosphere	D50 (μm)	Span	Grain Refinement
Ti-Al-Nb	3	Ar	233	1.56	Yes
	3	Air	191	1.72	Yes
	6	Air	163	1.50	Yes
	9	Air	113	1.67	Yes
	11	Air	48	2.10	Yes
Ti-Al-Ta	3	Air	56	2.03	Yes
	9	Air	54	1.79	Yes
Ti-Ta	1.5	Ar	70	1.62	No
	3	Ar	35	1.10	No
	6	Ar	22	1.17	No
	9	Ar	20	1.26	No

**Table 3:** Successful Isomorphic Inoculation Results.

Alloy	Grinding Time (h)	Grain Size (μm)	Equiaxed Fraction	Al Content (at %)
Reference		696	0.3	46.1
Nb Solutal		587	0.31	45.1
Ti-Al-Nb	0	403	0.69	45.6
	3 (Ar)	397	0.68	45.5
	3	336	0.63	45.5
	6	446	0.64	44.4
	9	337	0.64	45.4
	11	188	0.42	45.1
Ti-Al-Ta	3	335	0.52	45.7
	9	308	0.64	45.8

## References

[1] Kim Y.-W., Dimiduk D.M. (1991). Progress in the understanding of gamma titanium aluminides. JOM.

[2] Cowen C.J., Boehlert C.J. (2007). Comparison of the microstructure, tensile, and creep behavior for Ti-22Al-26Nb (At. Pct) and Ti-22Al-26Nb-5B (At. Pct). Metall. Mater. Trans. A.

[3] Witusiewicz V.T., Bondar A.A., Hecht U., Rex S., Velikanova T.Y. (2008). The Al–B–Nb–Ti system: III. Thermodynamic re-evaluation of the constituent binary system Al–Ti. J. Alloys Compd..

[4] Burgers W.G. (1934). On the process of transition of the cubic-body-centered modification into the hexagonal-close-packed modification of zirconium. Physica.

[5] Blackburn M.J., Jaffee R.I., Promisel N.E. (1970). Some Aspects of Phase Transformations in Titanium Alloys. The Science, Technology and Application of Titanium.

[6] Sankaran A., Bouzy E., Fundenberger J.J., Hazotte A. (2009). Texture and microstructure evolution during tempering of gamma-massive phase in a TiAl-based alloy. Intermetallics.

[7] Wu X., Hu D. (2005). Microstructural refinement in cast TiAl alloys by solid state transformations. Scr. Mater..

[8] Porter D.A., Easterling K.E. (2009). Phase Transformations in Metals and Alloys (Revised Reprint).

[9] Greer A.L. (2003). Grain refinement of alloys by inoculation of melts. Philos. Trans. R. Soc. A Math. Phys. Eng. Sci..

[10] Dantzig J.A., Rappaz M. (2017). Solidification.

[11] Hyman M.E., McCullough C., Levi C.G., Mehrabian R. (1991). Evolution of boride morphologies in TiAl-B alloys. Metall. Trans. A.

[12] Hyman M.E., McCullough C., Valencia J.J., Levi C.G., Mehrabian R. (1989). Microstructure evolution in tial alloys with b additions: Conventional solidification. Metall. Trans. A.

[13] Witusiewicz V.T., Bondar A.A., Hecht U., Zollinger J., Artyukh L.V., Velikanova T.Y. (2009). The Al–B–Nb–Ti system: V. Thermodynamic description of the ternary system Al–B–Ti. J. Alloys Compd..

[14] Hecht U., Witusiewicz V., Drevermann A., Zollinger J. (2008). Grain refinement by low boron additions in niobium-rich TiAl-based alloys. Intermetallics.

[15] Gosslar D., Günther R., Hecht U., Hartig C., Bormann R. (2010). Grain refinement of TiAl-based alloys: The role of TiB2 crystallography and growth. Acta Mater..

[16] Qian M. (2007). Heterogeneous nucleation on potent spherical substrates during solidification. Acta Mater..

[17] Cantor B. (2003). Heterogeneous nucleation and adsorption. Philos. Trans. R. Soc. A Math. Phys. Eng. Sci..

[18] Kuni F.M., Shchekin A.K., Rusanov A.I., Widom B. (1996). Role of surface forces in heterogeneous nucleation on wettable nuclei. Adv. Colloid Interface Sci..

[19] Kennedy J.R., Daloz D., Rouat B., Bouzy E., Zollinger J. (2018). Grain refinement of TiAl alloys by isomorphic self-inoculation. Intermetallics.

[20] Bermingham M.J., McDonald S.D., St John D.H., Dargusch M.S. (2010). Titanium as an endogenous grain-refining nucleus. Philos. Mag..

[21] Witusiewicz V.T., Bondar A.A., Hecht U., Voblikov V.M., Fomichov O.S., Petyukh V.M., Rex S. (2011). Experimental study and thermodynamic modelling of the ternary Al–Ta–Ti system. Intermetallics.

[22] Witusiewicz V.T., Bondar A.A., Hecht U., Velikanova T.Y. (2009). The Al–B–Nb–Ti system. J. Alloys Compd..

[23] Khallouk S. (2015). Developpement D’une Nouvelle Generation D’inoculants Pour les Aluminures de Titane. Master’s Thesis.

[24] Gale W.F., Totemeier T.C. (2004). Smithells Metal Reference Book.

[25] Huang S.C. (1993). Alloying considerations in gamma-based alloys. Structural Intermetallics.

[26] Shaffer P.T.B. (1964). Plenum Press Handbooks of High Temperature Materials: No. 1 Materials Index.

[27] Andersson J.-O., Helander T., Höglund L., Shi P., Sundman B. (2002). Thermo-Calc & DICTRA, computational tools for materials science. Calphad.

[28] Gibbs G.B., Graham D., Tomlin D.H. (1963). Diffusion in titanium and titanium-niobium alloys. Philos. Mag. J. Theor. Exp. Appl. Phys..

[29] Ansel D., Thibon I., Boliveau M., Debuigne J. (1998). Interdiffusion in the body cubic centered β-phase of Ta–Ti alloys. Acta Mater..

[30] Zollinger J. (2008). Influence de L’oxygene sur le Comportement a la Solidification D’aluminiures de Titane Binares et al. lies au Niobium Bases sur le Compose Intermetallique g-TiAl.

[31] Quested T., Greer A. (2004). The effect of the size distribution of inoculant particles on as-cast grain size in aluminium alloys. Acta Mater..

[32] Reilly N.T., Rouat B., Martin G., Daloz D., Zollinger J. (2017). Enhanced dendrite fragmentation through the peritectic reaction in TiAl-based alloys. Intermetallics.

[33] Gosslar D., Gunther R., Hartig C., Bormann R., Zollinger J., Steinbach I. grain refinement of gamma-TiAl alloys by inoculation. Proceedings of the Materials Research Society Symposium Proceedings.

[34] Quested T.E. (2004). Solidification of Inoculated Aluminium Alloys. Ph.D. Thesis.

[35] Hsieh T.E., Balluffi R.W. (1989). Experimental study of grain boundary melting in aluminum. Acta Metall..

[36] Straumal B.B., Gornakova A.S., Kogtenkova O.A., Protasova S.G., Sursaeva V.G., Baretzky B. (2008). Continuous and discontinuous grain-boundary wetting in Zn_x_Al_1−x_. Phys. Rev. B.

[37] Pereiro-López E., Ludwig W., Bellet D., Baruchel J. (2003). Grain boundary liquid metal wetting: A synchrotron micro-radiographic investigation. Nucl. Instrum. Methods Phys. Res. Sect. B Beam Interact. Mater. Atoms.

[38] Straumal B.B., Mazilkin A.A., Kogtenkova O.A., Protasova S.G., Baretzky B. (2007). Grain boundary phase observed in Al-5 at % Zn alloy by using HREM. Philos. Mag. Lett..

